# Improved fermentation efficiency of *S*. *cerevisiae* by changing glycolytic metabolic pathways with plasma agitation

**DOI:** 10.1038/s41598-018-26227-5

**Published:** 2018-05-29

**Authors:** Nina Recek, Renwu Zhou, Rusen Zhou, Valentino Setoa Junior Te’o, Robert E. Speight, Miran Mozetič, Alenka Vesel, Uros Cvelbar, Kateryna Bazaka, Kostya (Ken) Ostrikov

**Affiliations:** 1 Science and Engineering Faculty, Queensland University of Technology, Brisbane, QLD 4000 Australia; 2 Department of Surface Engineering and Optoelectronics, Jožef Stefan Institute, Ljubljana, SI-1000 Slovenia; 3 CSIRO−QUT Joint Sustainable Processes and Devices Laboratory, Commonwealth Scientific and Industrial Research Organisation, P. O. Box 218, Lindfield, NSW 2070 Australia

## Abstract

Production of ethanol by the yeast *Saccharomyces cerevisiae* is a process of global importance. In these processes, productivities and yields are pushed to their maximum possible values leading to cellular stress. Transient and lasting enhancements in tolerance and performance have been obtained by genetic engineering, forced evolution, and exposure to moderate levels of chemical and/or physical stimuli, yet the drawbacks of these methods include cost, and multi-step, complex and lengthy treatment protocols. Here, plasma agitation is shown to rapidly induce desirable phenotypic changes in *S*. *cerevisiae* after a single treatment, resulting in improved conversion of glucose to ethanol. With a complex environment rich in energetic electrons, highly-reactive chemical species, photons, and gas flow effects, plasma treatment simultaneously mimics exposure to multiple environmental stressors. A single treatment of up to 10 minutes performed using an atmospheric pressure plasma jet was sufficient to induce changes in cell membrane structure, and increased hexokinase 2 activity and secondary metabolite production. These results suggest that plasma treatment is a promising strategy that can contribute to improving metabolic activity in industrial microbial strains, and thus the practicality and economics of industrial fermentations.

## Introduction

To ensure long-term survival, organisms have evolved the ability to rapidly adapt to a wide range of highly variable environmental conditions^1–3^. Complex signal transduction networks allow cells to alter their basic structure, gene expression, cell cycle and cell-to-cell communication in response to external factors^4,5^. Different types of agitation may cause increased metabolic activity, promote cell growth and proliferation, or induce cell cycle arrest in the quiescent phase until external conditions are more favorable^6,7^. These adaptations can have significant consequences for cell-mediated industrial processes. For instance, in wine- and fuel-related fermentation processes, the phenotypic changes in response to increasing ethanol concentrations are considered a major cause for suboptimal fermentation, which for winemakers can lead to product downgrade or loss^8–11^ and limits productivities and therefore economics in industrial ethanol fermentations. Similarly, the availability of preferred, rapidly fermentable carbon sources such as glucose is a major determinant of cell cycle progression^12^, with nutrient depletion-related responses considered a significant limiting factor in biofuel production. Other environmental factors that can induce such intracellular signalling pathways include extreme temperatures, hydrostatic pressure, osmolarity, ultraviolet radiation, and reactive oxygen species^13^.

*Saccharomyces cerevisiae* is a well-studied unicellular eukaryote with a significant role in ethanol production and food fermentation^14^, as well as being a valuable model organism for common eukaryotic environmental response pathways. Although there have been many examples of improving yeast strains through rational and random genetic mutagenesis^15–20^, research to improve fermentation performance and enhance metabolic activity has also focused on pre-conditioning cells for increased performance through inducing phenotypic changes. Such responses involve a change in cellular activity to ensure survival, protect essential cell components, and to drive a resumption of cellular activities during recovery^3,5,7,21,22^. Tolerance to extreme environmental conditions is acquired by means of protective biochemical processes which include the synthesis of osmolytes (e.g. glycerol), trehalose, heat shock proteins (HSPs), increased chaperone activity, enhanced radical oxygen scavenging, changes in redox control, increased proton pumping activity, adjustments in carbon/nitrogen balance and altered ion and water uptake^6,23–27^. These response mechanisms not only initiate the repair of macromolecular damage caused by an environmental factor but presumably also establish a tolerant state, which helps prevent further damage. Central to these responses are the sensing and signalling pathways that communicate with the nucleus and facilitate necessary changes in gene expression.

Davies and co-workers^28^ showed that exposure to conditions under which yeast experiences a degree of disturbance in the balance between the production and neutralization of reactive oxygen species stimulates an adaptive response resulting in transient resistance to higher levels of the same conditions. The acquisition of tolerance to otherwise lethal levels of chemical and physical factors within a fermentation environment has been linked to an increased synthesis of proteins that mitigate these factors during pre-exposure treatment^29^, priming the cells to cope and respond more effectively as the environment becomes harsher. Such investigations suggest that yeast have an inherent ability to improve their fermentation resilience provided that the appropriate external and internal triggers are activated.

Furthermore, yeast cells exposed to moderately-hostile environments can develop tolerance not only to higher levels of the same environmental factor, but also to inhibitory activity caused by other agents. This phenomenon is called cross protection and is caused by the expression of general survival genes under moderately-challenging conditions^30^. For example, a brief temperature shock not only increases yeast thermal tolerance, but may also increase tolerance to ethanol^31,32^, high salt concentrations and oxidative damage^33^, to name a few. Steels and co-workers^34^ investigated the relationship between yeast tolerance to heat and oxidative damage, and found that a mild heat shock induced tolerance to an otherwise lethal temperature and H_2_O_2_ levels. Similarly, pre-treatment of yeast cells with a mild osmotic shock conferred increased resistance to heat shock^35,36^ and the exposure of yeast to ethanol, sorbic acid and low external pH induced greater thermo-tolerance^37,38^. This phenomenon of cross-protection is consistent with commonality in the yeast cellular responses and protection to different forms of environmental damage.

Although cross protection suggests commonality in survival responses, there is a level of exclusivity. For example, a mild heat shock was shown to have limited effect on osmo-tolerance^35,36^. Similarly, it has been shown that pre-exposure of yeast to low temperature conferred resistance to both low temperature and oxidative damage, but pre-treatment of cells with a low concentration of H_2_O_2_ did not evoke resistance to heat damage^34^. Thus, while a part of the survival response of yeast cells may be shared and lead to cross protection, there are also factor-specific responses.

We hypothesized that inherently complex environments, such as those afforded by non-thermal gas plasmas^39^, could provide a necessary degree of environmental agitation to precondition and thereby improve subsequent *S*. *cerevisiae* fermentation processes. Non-thermal plasmas are partially ionized gases which are generated by supplying energy to gaseous medium leading to dissociation of molecular bonds and ionization reactions^40,41^. Hence, plasma consists of positively and negatively charged ions, electrons as well as neutral atoms and molecules (e.g. radicals) and UV radiation^42^. Furthermore, due to the moderate gas temperature and the possibility of their generation at atmospheric pressure, the non-thermal plasmas can be used for direct treatment of living organisms with the aim to induce and select desirable changes at the phenotypic and/or genetic level^43–45^.

To date, plasmas have been primarily explored as the means to stimulate tissue healing, selectively induce apoptosis in cancer cells^46,47^, and for inactivation of a wide range of pathogenic microorganisms on artificial and living objects^48,49^. Studies conducted over the past decade concluded that effective inactivation of microorganisms is primarily attributed to plasma-generated highly reactive agents, including UV photons, reactive oxygen species (O_2_^−^, O_3_, O∙), reactive nitrogen species, charged particles, as well as electric field effects^50–52^. Among these effects, plasma toxicity is primarily linked to the activity of reactive oxygen species in a dose-dependent manner^53–57^. It is well-established that a short (e.g. 3–10 min) plasma treatment using kINPen has the capacity to induce increased ROS levels in a variety of prokaryotic and eukaryotic cells^42,58–60^, as well as a variety of other changes, e.g. physical damage of yeast cells and chemical modification of biomolecules^61^, where charged species were identified to play a major role^62^. Given the wide diversity of possible plasma-induced effects, the focus of this paper was to understand the biological implications of plasma treatment, e.g. upregulation/downregulation of specific enzymes and induction of pathways associated with conversion of simple sugars during ethanol fermentation. It has also been observed that at doses below that required to achieve complete inactivation, plasma treatment can markedly reduce the lag phase, and increase the specific growth rate of cells, thus shorten the exponential phase. However, since the focus of previous studies was primarily on plasma-assisted microorganism inactivation, the mechanisms underpinning the stimulatory effect of plasma agitation on microorganisms remain largely unexplored.

This paper aims to explore non-thermal atmospheric-pressure plasmas to deliver a highly dynamic, multi-component environment to drive phenotypic improvements in *S*. *cerevisiae*, specifically focusing on enhancing cell stability and ethanol production. By identifying molecular processes that underpin yeast responses to plasma agitation, it may be possible to develop a general protocol where plasma is used to controllably induce specific tolerance and modulate glycolytic metabolic pathways, enzymatic activity and secondary metabolite production in yeast. The principal mechanisms underpinning yeast acclimatization to plasma agitation remain under investigation, but greater knowledge in this field may enable the development of practices that will improve fermentation efficiency and reliability, thereby reducing costs to industry.

## Results and Discussion

### Plasma effect on yeast cells

An initial focus on improving yeast fermentation pointed to the importance of finding the right balance between length of plasma treatment and obtaining desirable effects on yeast cells.

**Figure 1:**
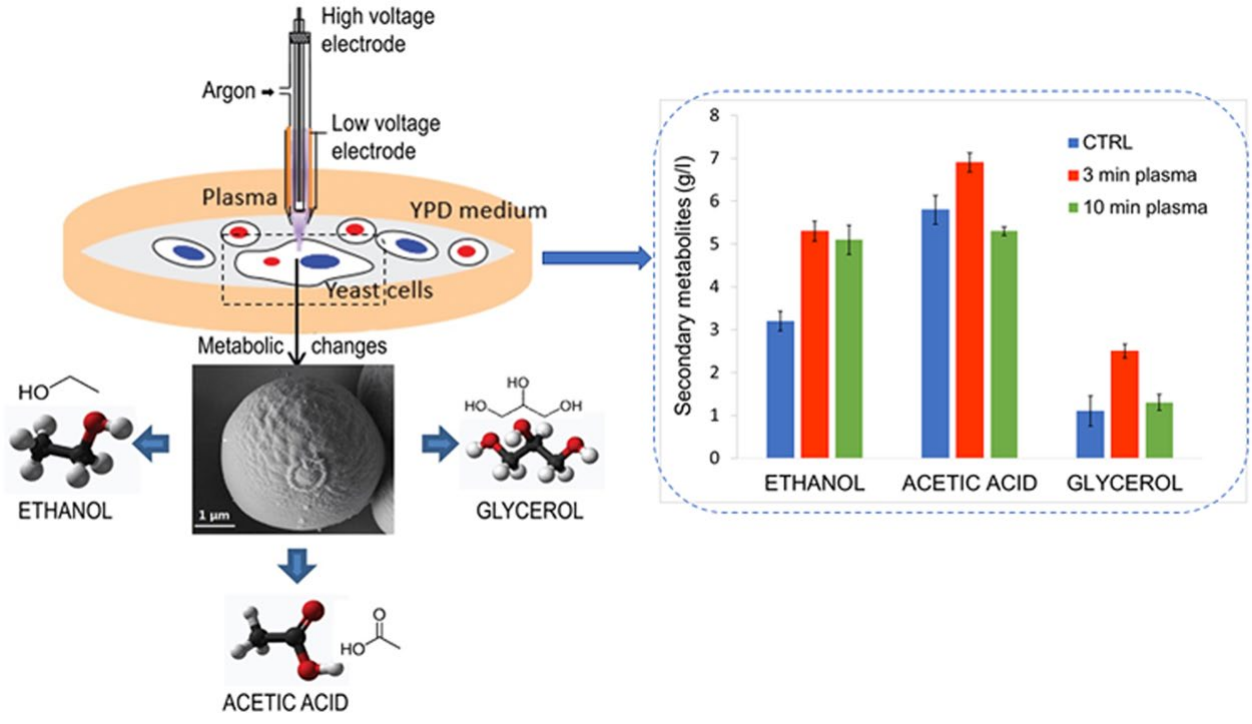
Strategy for plasma induced changes and metabolic pathway analysis in yeast.

We hypothesized that yeast cells subjected to plasma treatment could develop increased robustness and resilience and thus improve the efficiency of fermentation. Excessive doses of plasma treatment however have the potential to induce irreversible levels of damage or would kill a large fraction of the cells. We devised a strategy to treat yeast colonies with plasma prior to the entire colony being transferred to a starter culture media for expansion before inoculating a fermentation culture. The fermentation was then analysed for metabolites and enzyme activity (Fig. 1). This strategy was designed to evaluate whole of population phenotypic changes that resulted from plasma treatment of the colony rather than any single cell specific genetic changes.

The principles of plasma treatment involve combining reactive species that have different modes of action and different side effect profiles. Toxicity is therefore commonly observed with plasma reactive species and is dose-limiting^55,56^. Based on the plasma dose received, the severity and longevity of a plasma agitation on yeast cells is variable and so the response was accordingly attenuated.

### Short- and Long-term effects of plasma treatment on yeast cells

**Figure 2:**
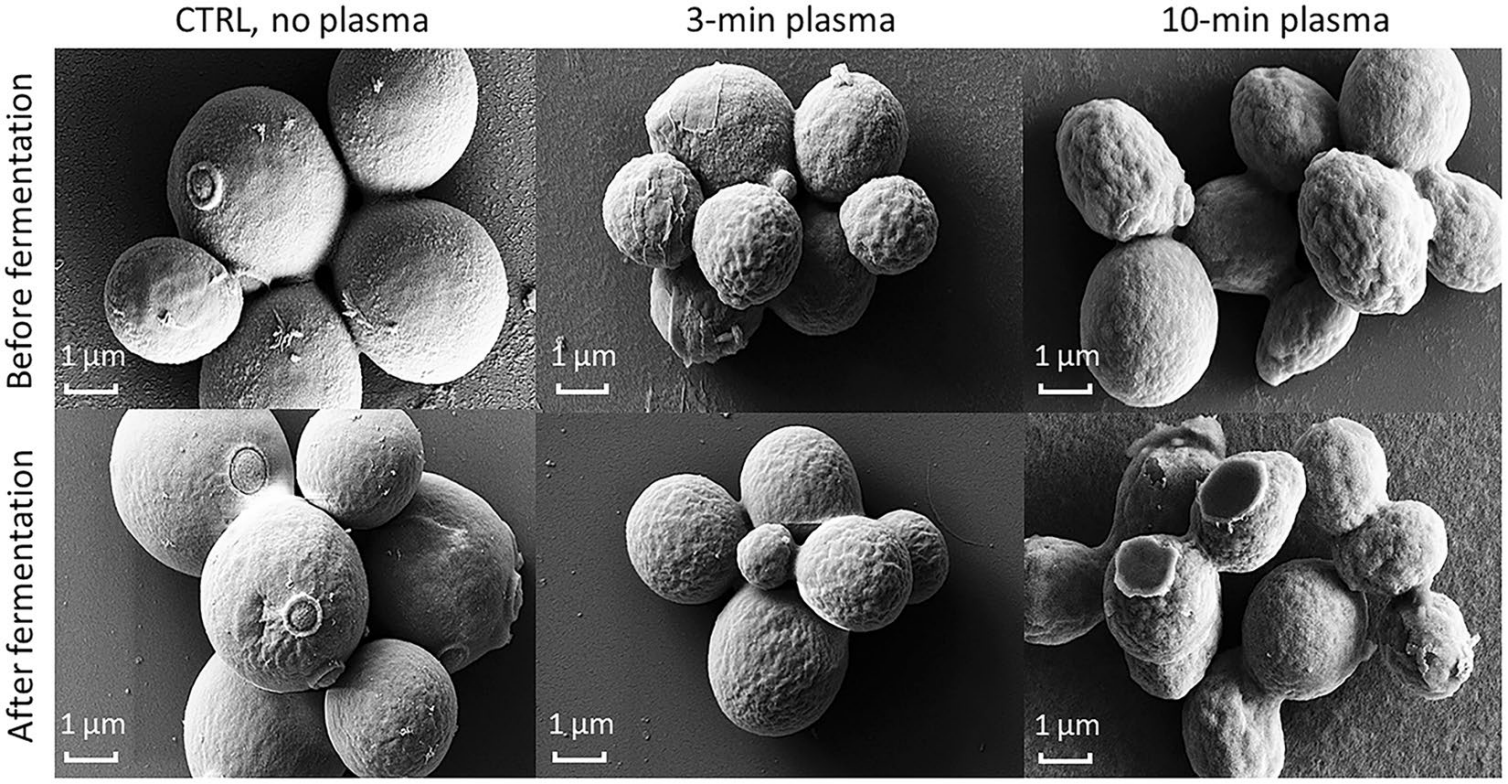
Representative SEM images of untreated and plasma-treated yeast cells. Changes in the characteristics of the cell membrane as a result of plasma treatment are retained from the colony treatment (before fermentation) to after the fermentation.

Yeast colonies were exposed to different plasma doses, from low (3 min of treatment), to medium (10 min of treatment), and ultimately lethal (20 min of treatment) doses of plasma. A large fraction of yeast cells were able to revert to normal growth once the plasma source was removed and cells were given an opportunity to recover. However, phenotypic consequences of plasma agitation, such as structural and possibly functional changes of cell membrane, may be retained by some cells after cell recovery. Plasma treatment-induced changes of cell membrane morphology and permeability of glial cells has already been reported previously^63^. Scanning electron microscopy (SEM) images of plasma treated yeast cells show the altered cell membrane when compared to untreated yeast cells (Fig. 2). From representative SEM images on Fig. 2, it is evident that the surface of *S*. *cerevisiae* treated with plasma is rougher, with the roughness increasing with increasing plasma treatment time. It has been previously suggested that *S*. *cerevisiae* cells may regulate the activity of transporters and other membrane proteins in response to exposure to osmotic and other types of stresses^64,65^ by changing spatial organization and dynamics of proteins in plasma membrane, and thus providing a specific lipid environment^66^.

Interestingly, plasma-induced changes to cell morphology were retained in the majority of the cell population harvested at the end of the fermentation despite these cells having gone through multiple cell divisions. These findings suggest that plasma treatment induces global long-lasting effects that remain long after the plasma exposure has concluded and are maintained throughout the population through cell division and growth. Given the population level effects these changes cannot be attributed to random genetic polymorphisms that would be specific to and different in each individual cell.

**Figure 3:**
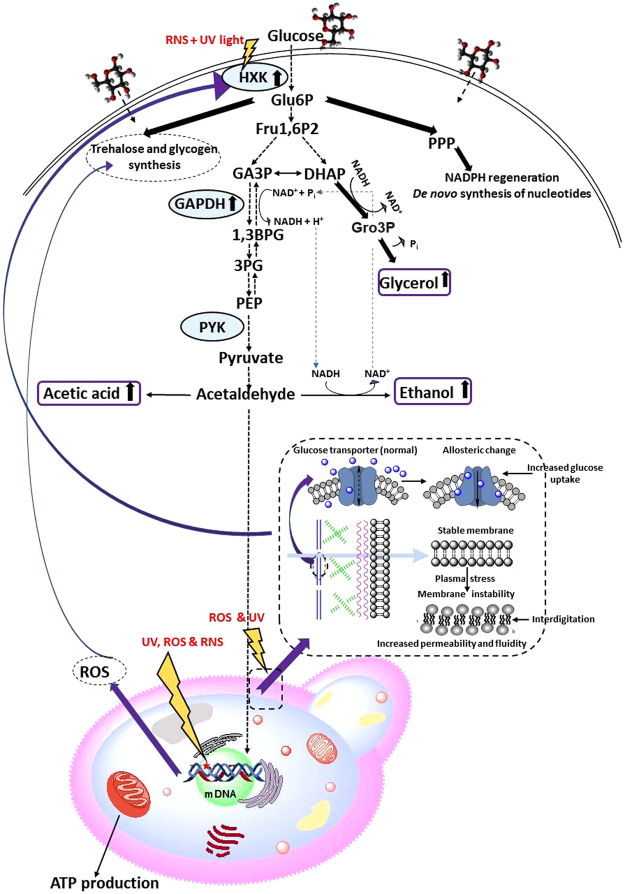
Schematic overview of glycolytic and fermentative pathways in plasma-treated yeast cells. The enzymes that were tested are shown in blue circles with an arrow representing upregulation after plasma treatment and absence of an arrow representing no observed change between treated or untreated cells. Similarly, the three tested metabolites are shown in purple squares. RNS: reactive nitrogen species; Hxk: hexokinase 2; Glu6P: glucose 6-phosphate; Fru1,6P2: fructose 1,6-bisphosphate; GA3P: D-glyceraldehyde 3-phosphate; DHAP: dihydroxyacetone phosphate; NAD^+^ and NADH: oxidized and reduced forms of nicotinamide adenine dinucleotide, respectively; GAPDH: glyceraldehyde 3-phosphate dehydrogenase; Gro3P: glycerol-3-phosphate; 1,3BPG: 1,3-bisphosphoglyceric acid; 3PG: glycerate 3-phosphate; PEP: phosphoenolpyruvate; PYK: pyruvate kinase; NADPH: reduced form of nicotinamide adenine dinucleotide phosphate; PPP: pentose phosphate pathway; RNS: reactive nitrogen species; UV: ultraviolet; ATP: adenosine triphosphate.

In nature, environmental factors rarely affect cells in isolation^67^. In laboratory or industrial settings, combinations of different factors can be made to interact synergistically or antagonistically to refine the response network or metabolism of the treated cell^68^. We found that in plasma-agitated yeast, glycolytic and fermentative pathways were modulated during subsequent fermentation. While certain enzyme activities were induced by the treatment conditions, it is also likely that certain pathways associated with cell survival may become modulated, with the effect of minimizing plasma-induced cellular damage and enhancing fermentation tolerance (Fig. 3). Importantly, an increase in secondary metabolite yield and productivity was observed in yeast treated with plasma (see Table 1). Although plasma agitation may induce random DNA single nucleotide polymorphisms in individual cells, we did not isolate single colonies post plasma treatment and we observed a global population effect. Accordingly, specific DNA mutations cannot account for the global population level effects we observed on the plasma treated yeast. If the underlying mechanisms responsible for the effects of plasma on yeast were understood, then this information could potentially be used to modify either the fermentation conditions or the genetic make-up of yeast strains to improve their fermentation performance.

**Table 1:** Concentration of secondary metabolites during 7 days of anaerobic fermentation using plasma treated and untreated yeast (0 day means the time of inoculation). Mean values (±Standard Deviation) for the respective triplicates are given.

	Fermentation time (days)							
Concentration (g/l)	**0**	**1**	**2**	**3**	**4**	**5**	**6**	**7**
**Ethanol**								
No PLASMA	0	0.65 ± 0.1	1.3 ± 0.1	1.6 ± 0.2	2.0 ± 0.05	2.8 ± 0.1	3.0 ± 0.4	3.2 ± 0.2
PLASMA 3 min	0	1.3 ± 0.2	1.8 ± 0.3	2.2 ± 0.1	3.0 ± 0.1	3.0 ± 0.2	4.2 ± 0.1	5.3 ± 0.1
PLASMA 10 min	0	0.75 ± 0.1	1.1 ± 0.2	2.0 ± 0.2	2.6 ± 0.3	3.2 ± 0.1	4.0 ± 0.1	5.1 ± 0.2
**Glycerol**								
No PLASMA	0	0	0	0	0.6 ± 0.2	0.75 ± 0.1	1.0 ± 0.1	1.1 ± 0.2
PLASMA 3 min	0	0	0	0	0.8 ± 0.2	0.9 ± 0.2	2.2 ± 0.3	2.5. ± 0.2
PLASMA 10 min	0	0	0	0.85 ± 0.2	0.95 ± 0.1	1.0 ± 0.05	1.1 ± 0.1	1.3 ± 0.1
**Acetic Acid**								
No PLASMA	0	0	0	2.2 ± 0.3	2.5 ± 0.4	3.6 ± 0.5	5.0 ± 0.6	5.8 ± 0.1
PLASMA 3 min	0	0	0	1.5 ± 0.4	2.5 ± 0.2	5.3 ± 0.7	6.1 ± 0.7	6.9 ± 0.4
PLASMA 10 min	0	0	0	0.8 ± 0.2	1.8 ± 0.5	3.35 ± 0.2	4.75 ± 0.6	5.3 ± 0.05

### Mechanism of action

Enzymes in relevant metabolic pathways during the fermentation were observed and modulation of native glycolytic pathways was studied and described in this paper. During the fermentation, samples were taken daily and the concentrations of ethanol, acetic acid and glycerol were determined. Concentrations of secondary metabolites were determined by GC–MS and the activity of two important glycolytic enzymes (hexokinase 2 and pyruvate kinase) was tested, using biochemical methods. Based on the results of secondary metabolites with GC–MS and enzyme activity, changes in glycolytic and fermentative pathways in plasma-treated yeast were observed (Table 1), suggesting modulation of metabolic pathways.

Hexokinase 2 (Hxk2) is the first enzyme involved in the metabolism of glucose. Upregulation of the activity of this enzyme may suggest induction by higher levels of glucose inside the cells^69,70^. The second enzyme studied in the glycolytic pathway is GAPDH, which is critical for glycerol production. The activity of this enzyme was shown to be upregulated. The activity of the PYK enzyme was also measured. PYK is responsible for pyruvate formation, which is then converted to ethanol via acetaldehyde. No up- or down- regulation was observed, although increased concentrations of ethanol were observed in plasma pre-treated yeast.

Hxk2 enzyme controls the very first step of glycolytic pathway, responsible for conversion of glucose to glucose-6-phospate. Hxk2 is also an evolutionarily conserved intracellular glucose sensor, which has been proposed to participate in sugar signalling and sensing in yeast^71^. Based on our results, the Hxk2 activity is strongly modulated by plasma treatment as observed by the evident increase in enzyme activity (Fig. 3).

As reactive oxygen species (ROS) and UV from plasma have been reported to lead to interdigitation of fatty acids in the membrane phospholipid bilayer and thus increased permeability of the cell membrane^72,73^, glucose uptake not regulated by glucose transporters may be affected. Furthermore, physical and chemical effects of plasma may potentially directly affect the activity of glucose transporters, i.e. major facilitator superfamily transporters, and thus facilitate enhanced glucose uptake. We have previously shown that plasma can change the chemistry of individual amino acids, affecting their function^74^.

Since activity of Hxk2 is related to glucose consumption from medium via glucose-induced suppression of the transcription of Hxk1 and Glk1 genes and upregulation of transcription of the Hxk2 gene^69,70^, and given the reported changes in cell permeability^75^, the increased intracellular concentration of glucose may subsequently lead to an increase in the rate of conversion of glucose to glucose-6-phospate and consequently enhanced glucose metabolism within the treated cells. It is also possible that the enzyme conformation and allosteric regulation of Hxk2 activity was altered by the nitrogen species from plasma (N^+^ and NO^−^) in combination with UV irradiation, thereby significantly affecting the glycolytic pathway.

**Figure 4:**
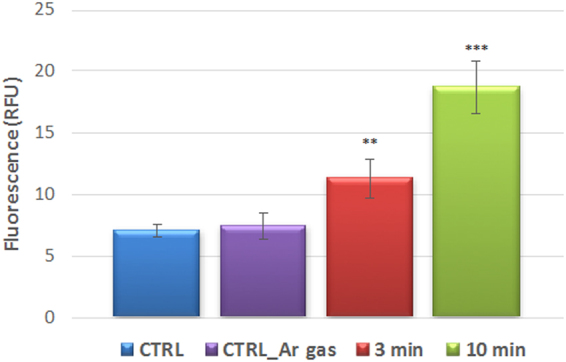
Oxidative stress response of yeast cells to different doses of plasma treatment. CTRL is cells not treated with plasma, CTRL_AR gas is cells treated with argon gas only and 3 and 10 min correspond to the length of plasma treatment. **Statistically significant at *p* < 0.01 compared with control. ***Statistically significant at *p* < 0.001 compared with control. Mean values (±standard deviation) for the respective triplicates are given.

The oxidative stress response of yeast cells to different doses of plasma treatment is shown in Fig. 4. Levels of intracellular ROS in live cells were detected using a fluorogenic probe, which emits bright orange luminescence once oxidized by ROS. Fluorescence corresponding to ROS concentration in cells is low in non-treated yeast cells and cells treated using Ar gas only, whereas in cells treated with plasma for 3 min, a moderate increase in intracellular ROS is observed, whereas the increase is more significant in the case of the 10 min plasma treated group.

**Figure 5:**
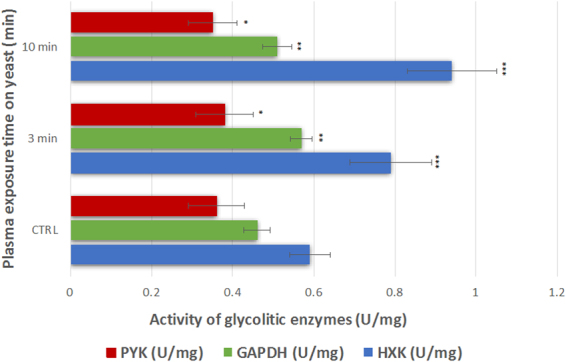
Activity of glycolytic enzymes Hxk2, GADPH and Pyk in plasma pre-treated (3 min and 10 min) and untreated control yeast (CTRL). **Statistically significant at *p* < 0.01 compared with control. ***Statistically significant at *p* < 0.001 compared with control. Mean values (±SE) for the respective triplicates are given. One unit of enzyme activity (U) is defined as the amount of the enzyme that produces a certain amount of enzymatic activity, that is, the amount that catalyzes the conversion of 1 micro-mole of substrate per minute.^76^

PYK is the enzyme which converts phosphoenolpyruvate to pyruvate and ADP. From here, decarboxylation of pyruvate to acetic acid is followed by further reduction to acetaldehyde and ethanol. Pyruvate kinase activity did not show any significant increase or decrease in activity in the plasma pre-treated yeast, when compared to the untreated control yeast (Fig. 5), however increased concentrations of ethanol were observed in plasma pre-treated yeast (Table 1) indicating that PYK does not limit flux through the pathway.

**Figure 6:**
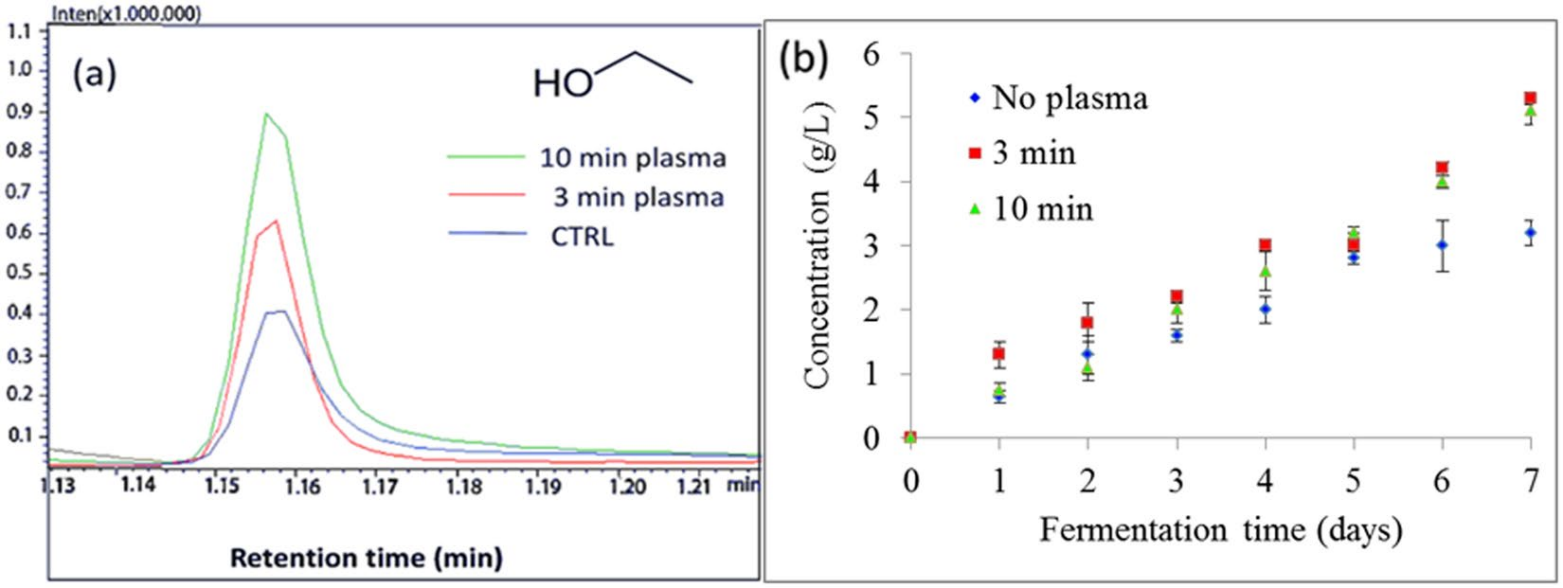
Ethanol production during 7 days of fermentation using parent yeast strain and strains resulting from 3 and 10 min plasma treatment. (**a**) Comparison of GC-MS analysis of ethanol, produced on day 7 of fermentation in non-treated control yeast group and 3 and 10 min plasma-treated group. (**b**) Final concentration of ethanol at each day of fermentation, analyzed by GC-MS. Statistically significant at *p* < 0.05 compared with control. Mean values (±SE) for the respective triplicates are given.

In our study the highest ethanol production was observed with plasma-treated yeast, during the stationary phase of growth, reaching 5.3 g/L on day 7 of the fermentation. The ethanol level in untreated yeast reached approximately 3.2 g/L on day 7 of fermentation, which is almost 40% less EtOH than in plasma-treated yeast (Table 1 and Fig. 6). Furthermore, the highest initial concentrations of EtOH were observed in 3 min plasma-treated yeast on the day 1, 2 and 3 of fermentation, assuming that 3 min plasma-treated yeast give the highest EtOH yield in the particular amount of time. One of the possible reasons for higher ethanol concentration in yeast may be an excess of NAD^+^ molecules in cells^77^. Exposure of *S*. *cerevisiae* to plasma agitation could prime glycolytic flux in pre-treated cells by regenerating NAD^+^ from accumulated NADH, which promoted reduction of acetaldehyde to ethanol, whereas the reduction of dihydroxyacetone phosphate to glycerol phosphate might helped to balance excess of NAD^+^ production, thus increased glycerol levels in plasma pre-treated yeast was observed.

**Figure 7:**
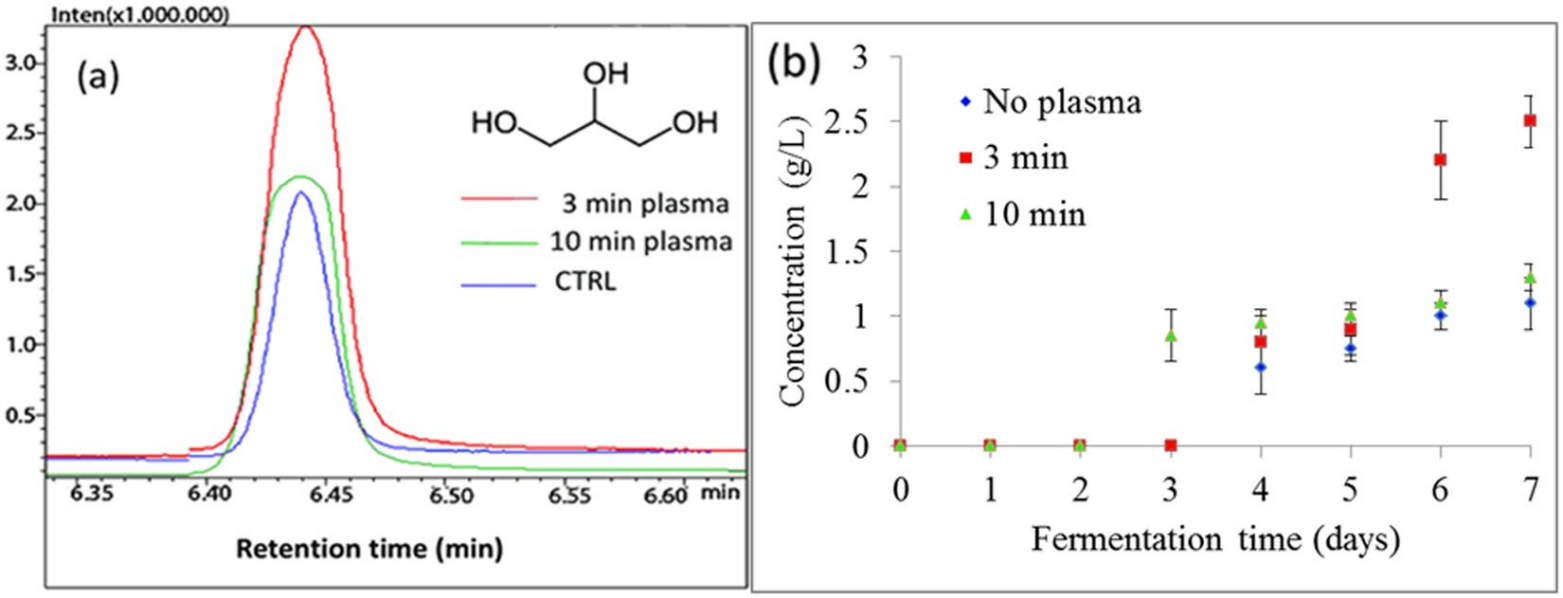
Glycerol production during 7 days of fermentation using parent yeast strain and strains resulting from 3 and 10 min plasma treatment. (**a**) Comparison of GC-MS analysis of glycerol, produced on day 7 of fermentation in non-treated control yeast group and 3 and 10 min plasma-treated group. (**b**) Final concentration of glycerol at each day of fermentation, analyzed by GC-MS. Statistically significant at *p* < 0.05 compared with control. Mean values (±SE) for the respective triplicates are given.

Substantial production of glycerol was observed during alcoholic fermentation in plasma pre-treated yeast. Glycerol was produced only on day 4 of the fermentation in non-treated and in 3 min plasma treated yeast, whereas in 10 min plasma treated yeast glycerol production was observed already on day 3 of fermentation and even in larger amounts than in the untreated cells. The majority of the glycerol was produced on day 6 and 7 of the fermentation, reaching values of 1.2−2.5 g/L in yeast treated with plasma for 3 min, whereas the lowest glycerol production was observed in the control yeast that did not receive any plasma treatment (Table 1 and Fig. 7). One of the possible explanations for increased glycerol production in plasma-treated yeast is that glycerol-3-phosphate dehydrogenase (GAPDH), the enzyme which converts glyceraldehyde-3-phosphate (GA3P) to 1,3-biphosphoglycerate (1,3BPG) is upregulated. This may be the reason why cells continue to accumulate glycerol as well as accumulate ethanol (see Fig. 3, Glycolytic pathways in yeast).

A possible reason for the increased glycerol was the high glucose level in the cells as a result of the alteration in Hxk2 enzyme activity. Another reason for increased glycerol production is an increased NADH concentration in cytoplasm, which is a consequence of decreased reduction of acetaldehyde to ethanol or alternatively, increased oxidation of ethanol to acetic acid. During buthic fermentation, it is believed that one of the main roles of glycerol formation is to equilibrate the intracellular redox balance by converting the excess NADH generated during biomass formation to NAD^[+ 78^. Overall, these effects on plasma pre-treated yeast increased the glycolytic rate.

**Figure 8:**
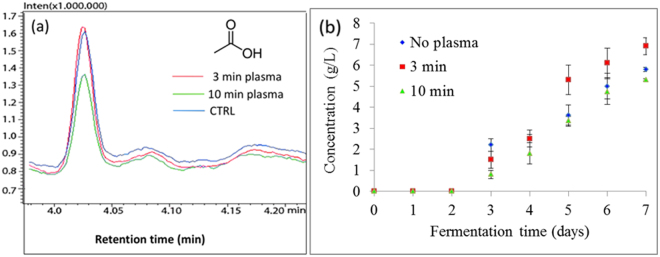
Acetic acid production during 7 days of fermentation using parent yeast strain and strains resulting from 3 and 10 min plasma treatment. (**a**) Comparison of GC-MS analysis of acetic acid, produced on day 7 of fermentation in non-treated, control yeast group and 3 and 10 min plasma-treated group. (**b**) Final concentration of acetic acid at each day of fermentation, analysed by GC-MS. Statistically significant at *p* < 0.05 compared with control. Mean values (±SE) for the respective triplicates are given.

Although the overproduction of glycerol in yeast cells that resulted from 3 min plasma treatment was observed, cell viability decreased, which was attributed to high acetic acid production during the stationary state of growth, between days 5 and 7 of fermentation (Table 1 and Fig. 8). Results of GC-MS analysis showed that acetic acid production differs between plasma-treated yeast and control group (Fig. 8). Production of acetic acid was observed only on day 3 of the fermentation, in both plasma-treated and non-treated yeast. On days 4 and 5 of fermentation the production of acetic acid was lower in plasma treated yeast in comparison to non-treated yeast, whereas more significant differences in concentration were observed from day 5 to day 7 of the fermentation. The highest acetic acid production was observed in yeast treated by plasma for 3 min, whereas the concentrations in the control group and that containing cells treated for 10 min were similar. The final concentration varied from ≈ 5.3–6.9 g/L (Table 1). 3 min plasma-treated yeast cells that produced the highest levels of acetic acid also produced the highest levels of glycerol.

**Figure 9:**
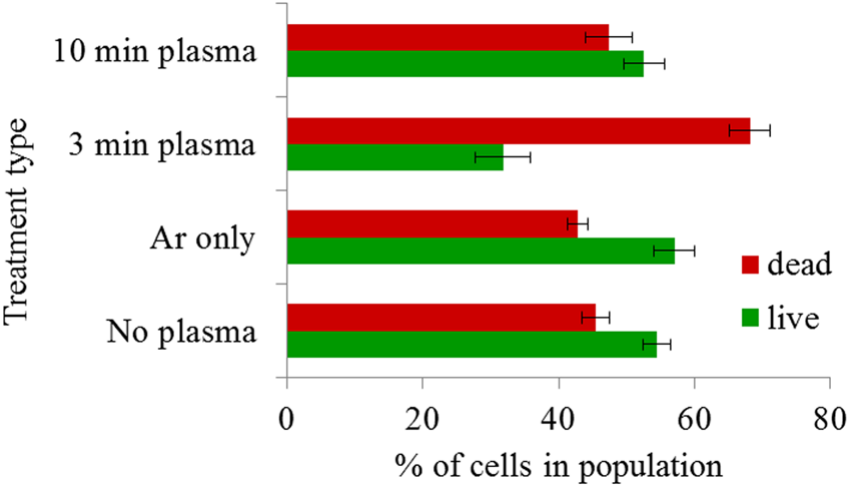
Viability of untreated and plasma-treated yeast cells after 7 days of fermentation. Statistically significant at *p* < 0.05 compared with control. Mean values (±SE) for the respective triplicates are given.

On the other hand, a strong decrease in cell viability was observed in 3 min plasma treated cells, indicating that those cells that survived the plasma treatment have managed to adapt with developing increased resistance and metabolite production. Similar cell viability was observed in yeast cells that were obtained as a result of 10 min plasma treatment when compared to the control, while cells resulting from 3 min treatment exhibited a strong decrease in cell viability (Fig. 9).

Immediately after plasma treatment, the cell viability of plasma-treated cell may decrease due to the ROS- and UV-induced damage, with some cells undergoing apoptosis. Numerous studies have reported that depending on the intensity and duration of the treatment, plasma agitation may cause oxidative damage in cells, resulting in cell de-homeostasis^79^, cell cycle arrest^54^ or/and apoptosis^50,80^. The viability of cells after 7 days of fermentation, on the other hand, would be mostly linked to the level of accumulated level of acetic acid, with cells treated for 3 min producing the highest amount of acetic acid (at 6.9 g/L after 7 days) compared to untreated cells (at 5.8 g/L) and cells treated with plasma for 10 min (at 5.3 g/L).

**Figure 10:**
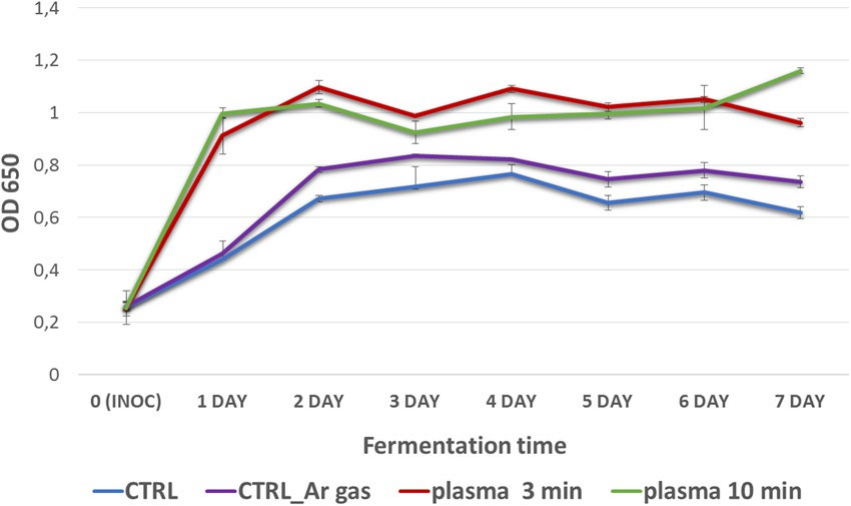
Growth curve of plasma pre-treated and non-treated yeast during 7 days of fermentation. Statistically significant at *p* < 0.05 compared with control. Mean values (±SE) for the respective triplicates are given.

Acting as a multimodal source of stress, plasma treatment stimulated faster yeast growth resulting in shorter exponential growth periods in comparison to control and higher final optical densities. It should be noted that immediately after subjecting actively growing yeast to plasma treatment, the rate of cell division may be reduced or even halted, with approximately 3 to 5 h of recovery time required for the growth to recommence. The culture containing inoculum treated with plasma for 3 min showed a 30% reduction in the exponential period compared to the non-treated (control) culture. When the effect of 10 min treatment was investigated, the exponential period was reduced by almost 60% (see Fig. 10). Thus, the plasma-induced exponential phase in yeast can be substantially reduced, suggesting stimulatory effects of plasma agitation of yeast that may operate by different and independent mechanisms.

As the consequence of the metabolic activity of the yeast cells, pH of the medium changed during the fermentation (see Supplementary Figure S1). By day 7 of fermentation, the medium containing cells treated with plasma for 3 min showed the lowest pH, which correlated with the highest level of acetic acid measured for this treatment group. The acidity of media in control group and the group containing cells that received plasma treatment for 10 min were similar. The curves representing CO_2_ released during the anaerobic fermentation in all three fermentations were of similar shape, although more CO_2_ was released in both fermenters containing plasma pre-treated yeast cultures (see Supplementary Figure S2).

Overall, plasma treatment evoked changes in yeast metabolic pathways and increased glycolytic rate during the fermentation^81,82^. These findings indicate that yeast cells continue to display the characteristics acquired as a result of plasma treatment long after exposure and responses are likely to operate by different and independent mechanisms. Previous studies have shown similar positive increase in performance in cells subjected to different types of stressors prior to fermentation. The enhanced performance was linked to stress response-related phenotypic changes, including changes in cellular activity likely to drive a resumption of metabolic and reproductive activity during recovery^3,5,7,21,22^. These processes include synthesis of proteins associated with stress protection, changes in membrane fluidity and conformation, increased chaperone activity, more effective removal of radical oxygen species, and alterations in redox control, and altered ion, glucose and water uptake^6,23–27^.

## Methods

### Plasma source and plasma treatment

For the experiments, plasmas were generated using a high-frequency (1.7 MHz, 2–6 kV), atmospheric pressure plasma jet (model kINPen08, Neoplas tools GmbH Greifswald, Germany) (Fig. 1). This plasma device consists of a pin-type electrode mounted in a quartz capillary (inner diameter of 1.6 mm and outer diameter of 4 mm) with an overall electric power of 65 W. A more detailed description of this type of plasma jet can be found in^83^. Argon (Ar) was used as feed gas at a gas flow of 5 standard liters per minute. The neutral gas temperature was kept below 40 °C to prevent thermal damage of the cells. The samples were located at a constant distance of 7 mm from the jet nozzle. At this working distance, the tip of the Ar plasma jet comes in contact with the substrate surface, whereas the Ar gas discharge plasma, with a total length of 12 mm, spreads and covers the entire area of the single colony, which is treated uniformly by plasma. Plasma treatment of cells and the exact position of plasma jet is seen on Supplementary Figure S3 showing experimental workflow. The single yeast colonies were treated for 3 or 10 min. The control samples were exposed to the gas flow without plasma ignition.

### Cell culture and plasma treatment

*S*. *cerevisiae* cells were cultured on YPD agar plates (Yeast extract, 10 g L^−1^; Bacteriological peptone, 20 g L^−1^, glucose, 20 g L^−1^; agar, 15 g L^−1^) and left to grow for 72 h before plasma treatment. Individual colonies were then subjected to direct plasma treatment optimized to prevent excessive heating or drying of the cells^84^. Immediately following the treatment, the colonies were transferred into 100 mL YPD media, and allowed to incubate at 28 °C for 18−24 h until sufficient cell concentrations were achieved. For fermentation, 100 mL aliquots of media with cell concentrations of approximately 2.5 × 10^6^ cells mL^−1^ were used to inoculate 0.5 L YPD fermentation media containing 10 g L^−1^ glucose. The parental wild-type strain of the yeast that was either not treated with plasma or treated with gas flow only were used as controls. Yeast cells were then cultured in 750 ml shake flasks fitted with fermentation locks (CO_2_ bubbling outlets filled with water). Fermentation was carried out under anaerobic isothermal conditions (30 °C) for 7 days in a shaker incubator at 90 rpm. Aliquots of 1 mL were aseptically withdrawn daily for the determination of cell number, dry weight and metabolite concentrations. At the end of the fermentation, the culture was centrifuged at 3000 rpm for 10 min, and the cell pellet and culture medium were stored separately at 4 °C prior to analysis.

### Microorganism and cultivation conditions

*S*. *cerevisiae* AWRI 1631 is a homozygous haploid strain, kindly provided by the Australian Wine Research Institute. Frozen stock cultures containing 40% (w/v) glycerol were stored at −80 °C. Working stocks were maintained on YPD agar slants (OXOID Yeast extract, 10 g L^−1^; OXIOD Bacteriological peptone, 20 g L^−1^, glucose, 20 g L^−1^; OXOID agar, 15 g) at 28 °C in the incubator remaining stocks, that were currently not in use, were stored at 4 °C in the fridge.

### Scanning electron microscopy (SEM) of yeast cells

Samples of untreated and plasma treated yeast cells were collected immediately after the plasma treatment, before inoculation and at the end of the fermentation process. Yeast cell morphology was assessed immediately after plasma treatment and after 7 days of the fermentation by scanning electron microscopy (SEM). Briefly, a drop of media containing yeast cells was put on a glass slide and fixed in 4% glutaraldehyde (Sigma-Aldrich, USA) in phosphate buffer solution for 30 minutes, followed by dehydration through an increasing gradient of ethanol and then vacuum dried by the critical point method. Finally, the samples were covered by a thin layer of gold using a Leica Gold Coater. SEM analyses were performed using a ZEISS Sigma Scanning electronic microscope under vacuum at 10–15 kV.

### Preparation of cell-free extracts

For preparation of cell extracts, the cell pellets were removed from storage at 4 °C, washed once with 100 mM potassium phosphate buffer (pH 7.5, 4 °C) and resuspended in 100 mM potassium phosphate buffer, pH 7.5, containing 2 mM MgC1_2_, and 1 mM dithiothreitol. The extracts were prepared immediately after washing by sonication of the cells with 0.7-mm diameter glass beads at 0 °C for 2 min using a MSE sonicator (150 W output, 8 pm peak-to-peak amplitude). Whole cells and debris were removed by centrifugation at 20,000 g (10 min at 4 °C). The clear supernatant, typically containing 2−4 mg protein mL^−1^ was used as cell-free extract.

### Protein determination

Protein concentrations in cell-free extracts were determined by the Lowry method. Dried bovine serum albumin (fatty-acid free, Sigma, U.S.A.) was used as a standard. The method is based on both the Biuret reaction, in which the peptide bonds of proteins and then reacts with copper under alkaline conditions to produce Cu + ions, which in turn react with the Folin reagent, and the Folin–Ciocalteau reaction. Briefly, phosphomolybdotungstate is reduced to heteropolymolybdenum blue by the copper-catalyzed oxidation of aromatic amino acids. The reactions result in a strong blue color, which depends partly on the tyrosine and tryptophan content. The method is sensitive down to about 0.01 mg of protein/mL, and is best used on solutions with concentrations in the range 0.01–1.0 mg/mL of protein^85^.

### Enzyme assays

Enzyme assays were performed using a Hitachi model 100–60 spectrophotometer at 30 °C with freshly prepared extracts. Reaction rates, corrected for endogenous rates, were proportional to the amount of extract added to the assays. When necessary, extracts were diluted in sonication buffer. The assays were performed at 340 nm (E_340 nm_ of reduced pyridine-dinucleotide cofactors = 6,220 M^−1^cm^−1^). Enzyme activities are expressed as μmol of substrate converted per min per mg of protein.

*Pyruvate kinase* (EC 2.7.1.40). The reaction mixture (1 mL) contained cacodylate buffer (pH 6.2), 0.1 mmol; KCl, 0.1 mmol; ADP, 10 μmol; fructose-l,6-diphosphate, 1 μmol; MgCl_2_, 25 μmol; NADH, 0.15 μmol; lactate dehydrogenase (Boehringer), 10 U; and 100 μL cell-free extract. The reaction was started by the addition of 10 mM-PEP.

*Glyceraldehyde-3-phosphate dehydrogenase* (EC 1.2.1.12) was assayed following the modified method by Bergmeyer [25]. The assay solution was produced by combing the cell extract with triethanolamine-HCl buffer (pH 7.6), 100 mM; ATP, 1 mM; EDTA, 1 mM; MgSO4, 1.5 mM; NADH, 0.15 mM; and phosphoglycerate kinase 22.5 U z ml^−1^ (Boehringer). The reaction was started with 5 mM 3-phosphoglycerate.

*Hexokinase 2* (EC 2.7.1.1). Hexokinase 2 activity was determined by measuring NADPH production at 28 °C in a reaction mixture containing: 50 mM imidazole buffer pH 7, plus 10 mM magnesium chloride, 25 mM glucose, 1 mM NADP, and glucose-6-phosphate dehydrogenase (1 U). The reaction was started by the addition of 2 mM ATP.

### Determination of intracellular ROS

Intracellular reactive oxygen species were quantified using CellROX® Orange Reagent (Life Technologies). *S*. *cerevisiae* yeast cells were treated with plasma for different time periods and plated in 96-well plates. The control group of cells, non-treated and yeast cells treated with Ar gas only were added to 96-well plate with 100 μl of YPD media/well. The cells were then stained with 15 μM of CellROX® Orange Reagent by adding the probe to the complete medium and incubating the cells at 37 °C for 30 minutes. The cells were then washed with PBS and analyzed using microplate fluorometry.

### Determination of secondary metabolites by gas chromatography−mass spectrometry (GC−MS)

Analyses of secondary metabolites were performed using a GCMS-TQ8040 Shimadzu (GC 8000 series, Model: 8040 MS: MD800). Samples of 1 μl were injected in a capillary column coated with CP-Wax 52 CB (50 m × 0.25 mm i.d., 0.2 μm film thickness; Chrompack). The temperature of the injector (SPI – septum-equipped programmable temperature) was programmed at 230 °C. The oven temperature was held at 35 °C for 2 min, then programmed to rise from 50 °C to 230 °C, at 4 °C/min. Helium at 103 kPa was used as carrier gas. The detector was set to electronic impact mode (70 eV), with an acquisition range from m/z 29 to m/z 400. Identification of volatiles was performed using the software from Wiley and Nist libraries, by comparing mass spectra and retention indices with those of pure standard compounds. Each determination was carried out in triplicate.

### Cell growth and viability

Cell growth was measured using a microplate reader. Growth was monitored over a 24 h period at 28 °C with the continuous orbital shaking and absorbance measured at 600 nm.

Cell viability was assessed by staining of cells with methylene blue. Cells were suspended in PBS, and a sample (100 μL) of the cell suspension was mixed with 100 μL methylene blue (0.1 mg mL^−1^ stock solution, dissolved in a 2% dihydrate sodium citrate solution) and incubated for 5 min at room temperature. Viability was examined under microscope from at least 200 cells in three biological replicates. Viable cells were colorless, and dead cells were blue.

### Fermentation kinetics

The amount of CO_2_ released was determined by measurement of culture weight loss every day during the fermentation process. The precision of weighing (±0.01 g) allowed calculation of the CO_2_ production rate with sufficient precision. The fermentation progress (FP) was calculated from the amount of CO_2_ released from the culture medium, according to the equation: FP = CO_2(t)_/CO_2(max)_, where CO_2(t)_ is the cumulative amount of CO_2_ released up to any time *t* and CO_2(max)_ is the cumulative amount of CO_2_ released during the whole fermentation. The use of FP instead of fermentation time allowed a normalization of fermentation kinetics closely linked to substrate disappearance from the medium.

### Statistical analysis

All the above experiments were performed in triplicates and independently repeated at least three times, unless otherwise stated. The results obtained are shown as the mean ± SE (standard error of the mean) for triplicates of cultures. The student t-test was used to test the effect different plasma treatments have on the fermentation yield and metabolic activity of yeast cells. A value of p < 0.05 was considered significant.

### Availability of data and materials

All raw data presented in this study are available from the corresponding author upon request.

## Conclusion

This work demonstrated that plasma agitation has the capacity to rapidly induce desirable metabolic changes in S. cerevisiae after a single brief treatment, leading to enhanced metabolic activity and, as a consequence, a faster and more efficient conversion of glucose to ethanol as well as higher secondary metabolite yields. Different exposure times produced a dose-dependent alteration of metabolic pathways: when compared to control, a significantly higher EtOH production was detected in 3 min plasma-treated yeast in the first period of fermentation and reduced levels of acetic acid were produced on day 3 and 4; glycerol in 10 min plasma-treated yeast was produced on day 3 and in higher concentration than in non-treated yeast on day 4. All these changes point to adaptation of yeast cells to plasma agitation through changes in metabolic activity.

Considering the demonstrated ability of plasmas to activate biomolecules and solutions^86,87^, plasma pre-treatment can be considered a promising stand-alone or auxiliary strategy that can contribute to improving economics of industrial fermentations. However, improved mechanistic understanding of molecular and biochemical bases for the observed metabolic changes in a wider range of microbial strains is required for optimization and translation of plasma-based processes into biotechnology.

## Electronic supplementary material

Supplementary material
