# Supplementary material for: Improved fermentation efficiency of S. cerevisiae by changing glycolytic metabolic pathways with plasma agitation
Source: Sci Rep. 2018 May 29;8:8252. doi: 10.1038/s41598-018-26227-5 (PMC5974074; doi:10.1038/s41598-018-26227-5)
Supplement: Supplementary file 1 — Supplementary material [file 41598_2018_26227_MOESM1_ESM.docx]

**Improved fermentation efficiency of *S. cerevisiae* by changing glycolytic metabolic pathways with plasma agitation**

Nina Recek^1,2^, Renwu Zhou^1^, Rusen Zhou^1^, Valentino Setoa Junior Te’o^1^, Robert E. Speight^1^, Miran Mozetič^2^, Alenka Vesel^2^, Uros Cvelbar^2^, Kateryna Bazaka^1,3*^, Kostya (Ken) Ostrikov^1,3*^

**SUPPLEMENTARY MATERIAL**

Supplementary Figure S 1: The level of pH in fermenters during the 7 days of anaerobic fermentation in plasma pre-stressed and non-stressed yeast.

Supplementary Figure S 2: Release of CO_2_ during the 7 days of anaerobic fermentation in plasma pre-stressed and non-stressed yeast.


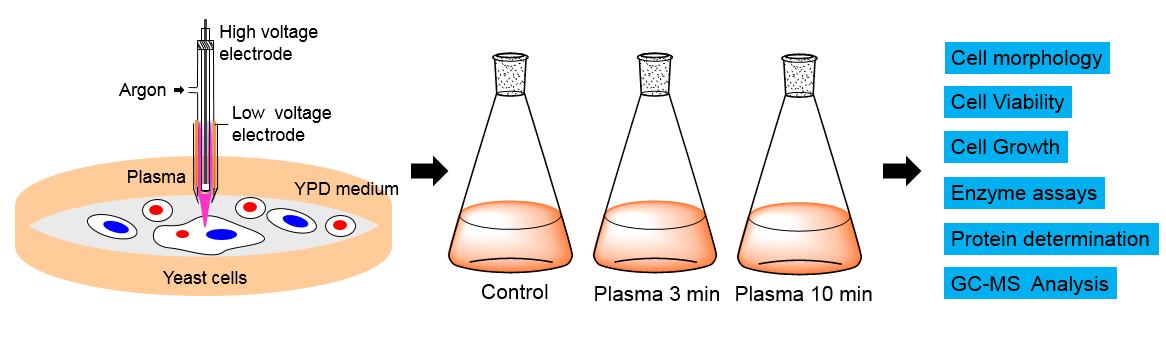


Supplementary Figure S 3: Experimental flow chart, showing the experimental steps.
